# Ecological Engineering: Reshaping Our Environments to Achieve Our Goals

**DOI:** 10.1007/s13347-012-0065-8

**Published:** 2012-01-27

**Authors:** Neil Levy

**Affiliations:** Florey Neuroscience Institutes, 161 Barry St, Carlton South, 3053 Australia

**Keywords:** Enhancement, Happiness, Psychology, Autonomy

## Abstract

Human beings are subject to a range of cognitive and affective limitations which interfere with our ability to pursue our individual and social goals. I argue that shaping our environment to avoid triggering these limitations or to constrain the harms they cause is likely to be more effective than genetic or pharmaceutical modifications of our capacities because our limitations are often the flip side of beneficial dispositions and because available enhancements seem to impose significant costs. I argue that carefully selected environmental interventions respect agents’ autonomy and are consistent with democratic decision making.

Despite the many and impressive achievements of human beings, there is a large and growing body of evidence that we are subject to important limitations as thinkers. We are irrational in a wide range of ways: our assessment of evidence is often badly skewed, we overlook facts that stare us in our face, we flip from one view to another based on irrelevant considerations, and so on. These limitations, once we come to grasp their extent and their depth, are truly dismaying. Moreover, their effects should not be underestimated: they are responsible for a wide range of problems in the lives of individuals, and in the broader society.

Increasing recognition of the limitations on our capacities naturally leads to consideration of what might be done to mitigate or correct for their effects. One tempting response to our limitations is the use of new technologies, perhaps genetic, pharmacological, or electrical interventions (genetic engineering, cognitive enhancement, or transcranial magnetic stimulation, for instance). The cognitive limitations of human beings are an unsurprising product of evolution. Evolution is a satisficing process, which tends to generate traits that are adequate to the challenges that confront organisms: given that the challenges we now face are of a kind, and a magnitude, that differs sharply from those we confronted in the environment of evolutionary adaptation, there is an apparent mismatch between our capacities and our needs. We might hope, therefore, to turn to technology to reshape our capacities so that they are more reliable and less susceptible to irrelevant influences. These responses to our limitations are especially tempting because they seem maximally *autonomy respecting*. They enhance capacities, leaving the decision about how best to utilize these capacities in the hands of individuals.

These kinds of technological solutions to the problem of our limited capacities may indeed be an option worth exploring. In this paper, however, I shall suggest that manipulating our environments might be a better solution to the problem. Rather than alter our onboard capacities, we should try to alter the environment to work with their imperfect grain, I shall suggest.

One reason to prefer social engineering—as we might call the kind of environmental manipulation I am going to defend here—to altering our onboard capacities is that the available means of altering our capacities are often messy: improvements in one of our capacities often cause deterioration in others. It remains possible that safe, effective, and cost-free enhancers might one day be developed. For the moment, however, it seems likely that we will be better able to counteract or mitigate the effects of our cognitive limitations by social engineering than by higher-tech solutions. The first aim of this paper is to defend this claim concerning the comparative effectiveness of environmental engineering and the direct enhancement of onboard capacities.

However, we ought to prefer ecological engineering to enhancement of our onboard capacities—or, indeed, to the status quo—only if the advantages of the former are not purchased at an unacceptable cost. The infringement of autonomy entailed by ecological engineering seems to be a cost that must be weighed against the benefits of increased capacities; other things being equal, maximally autonomy respecting interventions like cognitive enhancements ought to be preferred. The second aim of this paper is to argue that this apparent benefit of enhancement of our onboard capacities over ecological engineering is illusory. The latter is no less autonomy respecting than the former, I shall claim.

I will begin by reviewing evidence that the Enlightenment assumption that human reason is well designed to allow each of us to engage in the project that it called the pursuit of happiness is ill-founded. In fact, human beings, left to their own devices, are often remarkably bad at pursuing the goods they themselves endorse. I will then turn to sketching some proposals which would make us better at pursuing the goods we seek. I will end by arguing that these proposals are autonomy respecting, inasmuch as they enable us better to achieve aims we ourselves antecedently endorse.

## The Pursuit of Happiness

Liberals and libertarians believe that all restrictions on individual liberty, however minor, require justification. Typically (at very least) they set the bar high for such justification. Following Mill, they typically hold that the central justification for infringements of liberty is the prevention of harm to others. The infringement of liberty for any other reason, such as paternalistic intervention for the agent’s own good, is for some liberals and most libertarians entirely unacceptable; for others, it is justifiable only when the benefits clearly and greatly outweigh the harms. This animus against paternalism is a defining feature of liberal political thought.

Liberalism emphasizes liberty, in very significant part, in the name of the right we each possess to pursue our own conception of the good as we see it. Recognition of this right emerged for pragmatic reasons from the religious wars that racked Europe in the wake of the Reformation (Rawls 1993: xxvi). The alternative to finding a *modus vivendi—*a means of getting along with one another—was endless and ruinous war. But by the eighteenth century, the doctrine of tolerance for other ways of life was increasingly recognized as a *moral* principle. We have a *right* to pursue our own conception of the good life. Part of the justification for this idea comes from political philosophers pondering the purpose of the state. Many philosophers argued that the state existed only to allow autonomous individuals to pursue their own projects; since the state is constituted by the free adhesion of individuals, its legitimacy depends upon allowing each to pursue their projects without interference. It is precisely this doctrine that is expressed in the American Declaration of Independence: each of us has the “inalienable right” to the “pursuit of happiness” (as each of us sees it); the end of government is to secure these rights, so that when a government “becomes destructive of these ends, it is the right of the people to alter or abolish it.”

The right to pursue one’s own conception of the good without unjustified interference by the state or disapproving others is plausibly the central plank of liberal political philosophy. I will suggest, however, that liberals and libertarians have tended to set the bar for restrictions too high, by their *own* lights. We can and should restrict liberty *in order to enable individuals more effectively to pursue their own conceptions of the good*. Liberal political organization is rightly valued because it allows for the pursuit of rival conceptions of the good life, thereby respecting our autonomy. Because respect for autonomy is paramount in liberal societies, restrictions always require justification, and the more they burden or prevent choice, the better the justification must be. But a variety of restrictions can be justified, I shall argue, in the name of autonomy, rather than despite it. Insofar as the justification of liberal societies rests on their ability to allow a multiplicity of different conceptions of the good life to flourish, restrictions which make us better at pursuing our conception of the good, whatever it may be, do not genuinely conflict with the principles of liberalism.

Liberals and libertarians set the bar to interference with individual liberty too high due, in part, to an unrealistic view of human rationality. The Enlightenment, from which we inherited liberal principles, stressed the power of human reason to discover significant truths. The Enlightenment argued for the liberation of humanity from the constraints of traditional society, on the grounds that each of us is the best judge of our own good and of the means to pursue it. As Kant put it in his famous essay, Enlightenment is “man’s emergence from […] the inability to use one’s own understanding without the guidance of another” (Kant 1991, 54). All such enlightenment takes is “freedom to make public use of one’s reason in all matters” (Kant 1991, 55), for we are all equipped to reason our way to the good. It is this doctrine that underlies modern market economics: the distribution of goods in a market is optimal because it is responsive to people’s preferences. And it is the doctrine that underlies the centrality of informed consent and the animus against paternalism in contemporary philosophy and applied ethics.

The development of science was in some ways a spectacular vindication of Enlightenment faith in reason. However, this vindication was only partial: the social organization of science is central to its success, and this organization *requires* various restrictions on the participants. The success of science is not evidence of the power of unfettered human reasoning, but of human reasoning carefully channelled, through processes of peer review, control of entry into debates, and the distribution of cognitive labor. Without these restrictions, the picture is less bright, I shall suggest. On our own, we are relatively ill-equipped to use our reason in the central project bequeathed to us by the enlightenment: the pursuit of happiness. We are much less good than the Enlightenment thought at identifying the behaviors that will enable us to achieve the ends at which we aim, and at actually acting as we ourselves believe we ought.^1^

In the rest of this section, I will survey a small part of the evidence that we have far-reaching difficulties, without assistance, in acting in ways that are well designed to achieve the ends which we set ourselves. There is also plentiful evidence that we have severe limitations when it comes to choosing ends; that we are subject to a variety of cognitive biases that limit our ability to assess evidence and therefore raise the probability that the ends we set for ourselves will be based on false beliefs. For the most part, I shall ignore these limitations in our ability to set ends for ourselves, in favor of a focus on our ability to achieve our ends, whatever they happen to be.

The reason for this restriction is simple: there is reasonable disagreement about whether concern for autonomy requires us to respect people’s ends even when these ends rest, in important part, on the foundation of false beliefs. I aim to avoid this controversy by focusing only on interventions that allow people to pursue their own values and their own ends, whatever they may be, and which affect their beliefs as little as possible. Other thinkers who have advanced similar proposals to mine have justified interventions on the basis of agents’ well-being, and therefore offer a “welfare criterion” for interventions (see Loewenstein and Haisley 2008 for a review). As these thinkers recognize, these criteria, if they are adequate, justify genuinely, if moderately, paternalistic policies (“light” paternalism, in Loewenstein and Haisley’s phrase). Paternalism, even light paternalism, can be seen as an infringement of autonomy, but intervening to allow people to pursue their own ends cannot justifiably be seen as infringing their autonomy at all, I suggest.^2^

Human flourishing—eudaimonia—should not simply be identified with happiness. It may often be rational to sacrifice a large measure of happiness for other goals. However, for most of us under a wide variety of conditions, happiness is a significant component of flourishing. It is therefore disconcerting to discover that people are systematically bad at predicting what will make them happy. Consider, first, the phenomenon of *hedonic adaptation*: the way in which we tend to revert to our former level of happiness fairly quickly after major life events. People systematically overestimate the effect that life events will have on their happiness because they fail to take this phenomenon into account. Thus, for instance, most able-bodied people say that if they were to become disabled, they would be extremely unhappy; many think that they would no longer find their lives worth living. But after actually becoming disabled people adapt; they return to a level of happiness that often does not differ significantly from the level of well-being they experienced prior to disability. One week after experiencing a disability negative emotions outweigh positive, but as soon as the eighth week the subjects report a preponderance of positive emotions (Silver 1982). The same phenomenon, in the reverse direction, occurs after positive life events such as winning the lottery (Brickman et al. 1978).

More recently, evidence has accumulated that suggests that the initial enthusiasm for hedonic adaptation exaggerated its extent. There is now strong evidence that “set point” theory, according to which people have a fixed (perhaps innate) happiness level that is impervious to life events, is untenable in its strongest form. Life events can indeed raise or lower happiness levels; indeed, they can raise or lower our set point, such that we become resistant to further life events, but at a different happiness level (Diener 2008). This entails that it is not futile to attempt to pursue happiness, nor to guard against averse life events like disabling accidents in order to preserve happiness (quite apart from the impact such event have on other measures of well-being). It remains true, however, that the impact of life events is often far smaller than individuals predict.

Locked-in syndrome (LIS) presents us with a dramatic illustration of hedonic adaptation. LIS is a state of almost total paralysis following a stroke; at *most*, sufferers have voluntary control only over the ability to blink. Many cases of LIS are misdiagnosed as persistent vegetative state, in which higher brain function, and probably consciousness as well, is lost. But in LIS the person is intact: they are looking out from within a shattered body. The lucky ones are able to communicate using their eye blinks. Some have even been able to use interfaces that connect them to computers, giving them the ability to use the internet and send email. Nevertheless, when we think what it must be like to be locked in, we seem presented with an image of unmitigated horror.

But this view seems to be mistaken. The phenomenon of hedonic adaptation ensures that things are not nearly so bleak. Bruno et al. (2008) asked normal controls and LIS sufferers to construct a personalized well-being scale, with −5 on the scale corresponding to the time in their life at which they were most unhappy and +5 corresponding to the time at which they were happiest. Subjects were then asked to rate the most recent 2 weeks of their lives using their personalized scale. Bruno et al. (2008) found that normal controls rated their past 2 weeks at an average of around 2. So, surprisingly, did sufferers from LIS. It should be noted, however, that the standard deviation was much higher for the latter group than the former. That is to say, although the average was about the same, there was much more variety among the LIS patients than the controls. Some sufferers from LIS really do rate their well-being very low, but many are sufficiently happy to bring the average up to around the same levels as controls. Hedonic adaptation is a powerful force.

Lack of knowledge of the power of hedonic adaptation ensures that people are poor *affective forecasters*: they have difficulty predicting the impact that events will have on their happiness. They therefore make bad choices, insofar as they aim to promote their own happiness. For instance, looking at revealed preferences shows a strong preference for income over other goods. This is prima facie evidence that people believe that higher income will lead to higher levels of subjective well-being. There is indeed a positive correlation between income and subjective well-being—richer people are, on average, happier than poorer—but the relationship is weaker than people seem to think. First, the relationship between higher incomes and higher subjective well-being is in part the result of higher subjective well-being leading to higher incomes, and not the other way round (Diener and Biswas-Diener 2002). Second, and more importantly, above a certain threshold rising incomes are subject to fast diminishing marginal returns. Inability to meet one’s basic needs has a significant effect on subjective well-being, but above that threshold, higher incomes have little effect (Myers and Diener 1995). Moreover, the pursuit of happiness via the pursuit of greater income tends to undercut itself. First, though it is true that relative income makes a difference, the effects of relative income on subjective well-being quickly diminish (though not to zero). The reason for this is apparently that as incomes rise, the reference group to which we compare ourselves changes. So, changes to happiness caused by changes in relative income tend to dissipate (though much less for people who do not place themselves in situations in which comparative assessments with a new reference group become probable). Second, and regardless of the ways in which we are led to change our reference groups as a consequence of a rise in relative income, pursuing happiness by pursuing income is a self-defeating project when it is broadly engaged in. One reason, of course, is that rises in income are inflationary pressures, but even rises in real income are self-defeating, inasmuch as above a certain threshold it is relative income that matters. This is an instance of the consumption treadmill (Sunstein 2007), where we have to run fast just to keep up.

Unsurprisingly, then, rising incomes in the wealthy societies have not caused an increase in happiness. In fact, there is some reason to think that happiness is actually falling (Haybron 2007). Consider the incidence of depression, which is rising in all industrialized countries. The gap between revealed preferences and the effective means to well-being suggests that people are not very good at making important decisions. Assuming (very plausibly) that people aim to increase their happiness, they are doing a bad job at it.^3^ They are working harder, and having much greater environmental impacts (thereby increasing the probability of a precipitous fall in well-being down the track) for little or no near-term gain.

Our inability to predict what will make us happy has adverse consequences for others, as well as for ourselves. At least, that is one possible interpretation of recent work on revenge. Carlsmith et al. (2008) had subjects play a bargaining game in which if everyone cooperated, all subjects benefited. Confederates of the experimenters defected and benefited disproportionately. The experimenters then gave some of the subjects a chance to punish the defectors: they could spend some of the money they had earned in the experiment to make defectors worse off. Virtually all subjects offered the opportunity to punish defectors took it. Why? Subjects who did not get the opportunity to punish were asked how they would have felt if they had been able to punish defectors; they said they would have been happier. So, it is plausible that those who did punish were motivated (in part) by the same belief. The belief was in fact wrong: subjects who did not have the opportunity to punish were happier than those who had punished. But subjects who had punished were not aware that they had a lower level of subjective well-being *because* they had punished: they predicted that they would have felt even worse had they refrained from punishing.

In many cases, the phenomenon of hedonic adaptation causes agents to be satisfied with suboptimal outcomes. In these cases, it might arguably be held to be paternalistic (objectionably or not; I take no stand on the issue here) to attempt to mitigate its effect. In some circumstances, however, hedonic adaptation will give rise to regret: when agents choose actions in the expectation that they will lead to a substantial and long-lasting rise in their happiness levels. This regret gives us the justification for interventions that do not compromise autonomy since they are consistent with (rather than seeking to change) the agent’s own existing values and deepest beliefs

Moreover, our incompetence at affective forecasting and inability to choose goods or courses of action that will make us happy is not an anomaly. Social and cognitive psychology has accumulated plentiful evidence that we are unskilled at making choices that correspond to our own deepest values. Consider hyperbolic discounting (Ainslie 2001). It is rational to discount future goods; that is, to think that the opportunity to secure future access to a good is worth less than the opportunity to have immediate access to the same good. For instance, if I offer you a dollar now or $2 in 3 months time, you might rationally prefer to take the dollar now. This might be the rational choice for any of several reasons: because you cannot be certain to get the money in the future (I might be untrustworthy; you might die in the interim) or because you expect to have less need of the money then than now. But hyperbolic discounting does seem to be irrational; certainly, it often interferes with the ability of agents effectively to pursue their goals. Agents discount future goods hyperbolically when their discount function is itself sensitive to the imminence of opportunities for consumption of goods. Hyperbolic discount curves can cross, and therefore the preferences of hyperbolic discounters can be highly unstable. Hyperbolic discounters experience preference reversals of the following sort: asked on Monday whether they prefer $1 on Tuesday or $2 on Wednesday, they might choose to wait until Wednesday and take the $2. But if they discount the future hyperbolically, as the opportunity for consumption gets closer, their valuation of the nearer good increases disproportionally. On Tuesday, they may value immediate consumption more than waiting the extra day, even when they know that taking it will preclude the larger reward. Hyperbolic discounters therefore experience preference reversals, followed by regret over lost opportunities for larger rewards.

Hyperbolic discounting explains many failures of prudential rationality. It almost certainly plays a role in drug addiction and other kinds of addictive behavior; it also helps to explain one of the greatest public health problems facing Western nations today: the obesity epidemic. People who overeat—and that is, to a first approximation, all of us—generally value health more than they value cheeseburgers, but they find their preferences temporarily shifting when the opportunity for consumption presents itself. Predictably, they come to regret their actions, and the cycle begins again.

Worst of all, we are subject to a variety of *positive illusions*: beliefs that we are more competent in key areas than we actually are (the more we value a skill, the higher the likelihood that we will attribute it to ourselves). These positive illusions may be psychologically beneficial, even necessary; perhaps, as Elster (1983) has suggested, we will only be sufficiently motivated to take on difficult tasks if we believe we are more likely to succeed at them than other people. Whatever the explanation, these positive illusions are comically pervasive: 80% of drivers judge themselves to be in the top 30%; most students judge themselves to be more popular than average; a full 94% of university professors believe they are better-than-average at their jobs (Gilovich 1991, 77), and so on. Only depressed people seem to have relatively accurate views of themselves (“depressive realism”; Alloy and Abramson 1979).

Now, while these positive illusions may be benign in many situations, and even beneficial in some, they often have deleterious consequences for our decision making. They work in concert with our myopia for the future, reflected in hyperbolic discounting, to cause imprudence. As Robert Frank (1999) has pointed out, in addition to the millions of Americans without health insurance because they cannot afford it, there are many millions without it who could afford it, and do not take it out: the propensity to believe that one’s risk of serious illness or accident is lower than average surely plays a role here. Positive illusions also probably play a role in persistent undersaving for retirement, for example.

Moreover, positive illusions interact with our other biases in ways that make them worse, and harder to correct for. Since we are subject to pervasive positive illusions, we are far more confident in our judgments than we ought to be (Fischoff et al. 1977); this result has been found to hold true across a wide range of tasks. This overconfidence, coupled with a resistance to accepting that our judgments are affected by the psychological mechanisms just outlined (which are, it must be stressed, a small sample of the psychological causes of bad choices), makes correcting for these biases very difficult. Subjects do not see the need to correct for their biases. Even when the experimental literature is pointed out to them, they remain confident that their judgments are objective. Subjects who accept that the experiments demonstrate the pervasive existence of irrationalities remain convinced that they themselves are not subject to them. This makes the application of what is known as *debiasing—*the implementation of strategies to compensate for biases—exceedingly difficult.

Moreover, it is not only laypeople who are subject to overconfidence. Genuine experts, those whose judgments in a particular domain really are much better than average, nevertheless vastly overestimate the reliability of their judgments. This overconfidence has been found to severely limit the effectiveness of measures taken to improve human reasoning. For instance, in a number of domains statistical prediction rules—rules which weigh various factors and generate a prediction—have been found to outperform expert judgments. Yet experts either refuse to implement such rules or ignore their results when they are implemented. Gladwell (2005: 136–141) gives the example of a relatively simple prediction rule that outperforms experienced physicians on the task of assessing the likelihood that a patient suffering chest pains is having a heart attack. Doctors resisted the implementation of this algorithm in emergency wards. Even after having accepted that it was generally accurate, moreover, they overrode its judgments in cases in which they considered it was obviously wrong. Yet in the majority of cases in which the attending physician concluded that the algorithm had clearly generated the wrong result, it was the physician that was wrong. This is a common type of finding: even when we are helped by statistical rules, and even when we accept that they are reliable, indeed, even when we are told that other experts who judged that the statistical rule had obviously got a case wrong were more likely than not to be wrong themselves, we are still more confident in our own judgment than we ought to be (Bishop and Trout 2005).

In this section, I have sketched a small part of the evidence that human beings are subject to a range of cognitive distortions, and volitional pathologies, which make us less good at achieving our goals than is widely believed. In the next, I will consider some proposals designed to make us better at achieving our goals.

## What Is to Be Done?

How should we respond to evidence of our cognitive limitations, and the ways in which they interfere with our capacity to achieve our ends? Despite the problems caused by overconfidence, debiasing can sometimes be successful. Take the confirmation bias, which refers to our propensity to look for evidence in favor of a hypothesis we are considering and overlook evidence against it (Nickerson 1998). This is frequently harmful. For instance, therapists of various sorts often seem to believe their favorite theories as a result of the confirmation bias. They think that, say, patients recover from distress by being encouraged to recover repressed memories of sexual abuse—despite the fact that there has never been a verified case of someone recovering a repressed memory of abuse—because they can recall instances of patients with whom they tried the therapy and who subsequently seemed to improve. They fail to recall, or to give due weight to recalling, all those patients who did not improve after the therapy (who may even have got worse), or they ignore the base rate of improvement regardless of therapy. There is evidence that debiasing strategies are effective against the confirmation bias: if you remind yourself of the need to conduct symmetrical memory searches, you are less susceptible to the effect (Lilienfeld et al. 2009). But there are limits to what can be achieved through debiasing. As we have already seen, debiasing is effective only for agents who believe that they need it. Those therapists (for instance) who are convinced of the truth of their theory simply dismiss the need to debias themselves. Second, personal debiasing strategies—techniques that agents can apply themselves—are costly and time-consuming to implement. But most of the time in most situations, our judgments are not made by conscious deliberation (Bargh and Chartrand 1999), which greatly limits our opportunities for implementing such strategies, even if we can be convinced of the need to do so. Moreover, we can implement debiasing strategies only if we recall the need then and there, are motivated to use the appropriate strategies, and if we are aware of the direction and magnitude of the bias (Trout 2005). These are demanding conditions. Moreover, we need to have available sufficient cognitive resources to implement the strategies. Conscious reasoning relies upon a depletable resource (Levy 2011a). All of us will frequently find ourselves with insufficient cognitive resources to overcome our biases.

Hence, correcting for the effects of the biases to which we are subject by way of personal debiasing is likely to be ineffective. What is to be done? I suggest that environmental engineering is an important part of the answer. We ought to implement social policies which shape our environments so that our cognitive weaknesses are dampened and our strengths enhanced. Intelligent social engineering can compensate for our ineptitude as affective forecasters and for our tendency to discount hyperbolically.

Bad affective forecasting will lead us to make choices that result in our having lower levels of subjective well-being than we might have had. Improving affective forecasting, or circumventing its ill effects, might therefore play a role in leading people to better decisions or (at least) better outcomes. This gives us grounds for interventions that might be seen as paternalistic, but which aim not (merely) at improved welfare, but at autonomy enhancement: interventions that aim at enabling agents better to achieve the ends *they* endorse. Firstly, it gives us reasons in favor of directive counseling. There is some evidence that drawing the attention of subjects to the phenomenon of adaptation is effective in making estimates of the hedonic impact of life events more realistic (Ubel et al. 2005); insofar as agents aim at happiness, this intervention may be autonomy enhancing. But this method has all the problems endemic to debiasing. Hence, we might opt for something more directive, and (apparently) more paternalistic, such as making options that predictably lead to worse outcomes less accessible (without actually preventing access). For instance, there are many thousands of people who believe that gender reassignment surgery will make them significantly happier. There is some reason to believe that they are wrong (Batty 2004). The evidence is not yet conclusive, but if and when it becomes strong enough, there may be a good case for making the surgery less accessible. We can do this without banning it; we can make the barriers to access higher (not financially, which would be discriminatory, but—for instance—by requiring very lengthy counseling, some of it aimed at attempting to convince the person that they ought not to have it).

The adverse effects of hyperbolic discounting, too, can be avoided by limiting people’s options without actually removing them. Hyperbolic discounting occurs when the opportunity for consumption of goods is imminent. Hence, we can better ensure that people make choices in line with their *own* values by limiting their opportunities for consumption; that is by ensuring that opportunities for immediate consumption of tempting goods but which, all things considered, they prefer not to consume, are less frequent. This would be impracticable if there were a very wide variety of goods in this class, with different goods being tempting for different people, but fortunately goods like this are relatively few. They fall into two rough categories: goods which we are adapted to find tempting because in the environment of evolutionary adaptation these goods were scarce and essential, but which today are easily available (calories, in particular) and goods that hijack neural pathways which are responsive to goods belonging in the first category (drugs and alcohol, in particular).

How do we control access to these goods in a way that avoids hyperbolic discounting, without violating autonomy? Hard line methods, which aim at preventing access altogether (prohibition), may not sufficiently respect autonomy. Though without any restrictions on access to alcohol (say), agents may regularly find themselves drinking more than they intended or in ways they later regret, these same agents may genuinely value the opportunity to consume more moderately, and some few may genuinely value heavy consumption. Fortunately, softer line methods, which limit access without removing it, may enable both groups to engage in the behaviors they genuinely value while reducing the extent to which either acts in ways they later regret. Softer line methods include restricting the hours of opening of bottle shops and other outlets for the sale of alcohol or weighing the options to make the likelihood of discount curves crossing smaller. Restricting the sale of alcohol helps to ensure that agents do not buy it when they are already intoxicated or when their cognitive resources are depleted for some other reason (tiredness for example), while allowing those who genuinely value all-night drinking to pursue this activity, by buying the alcohol beforehand. Weighing the options can make consumption costlier—through taxation, for instance—thereby reducing the likelihood that when opportunities for consumption are imminent, agents’ discount curves cross, leading them to act in ways they will later regret. Further, we can design environments to avoid depleting agents’ self-control resources, for example by reducing the likelihood that they will encounter temptations in too rapid a succession. Laws that govern the placement and content of advertizing, and laws governing the density and number of outlets selling tempting goods—alcohol, fast food, or what have you—can, if well designed, allow agents to manage their cognitive resources better. These measures make it more likely that on each occasion of temptation, agents choose in accordance with their own values, while allowing those with unusual values to continue to pursue them.

I have suggested that social engineering which does not violate autonomy but enables us to better achieve goods we value is feasible. I shall now argue that such engineering is likely often to be preferable to pharmaceutical or technological modification of our onboard capacities, affective, moral, or narrowly cognitive, for the achievement of the same end. Though such modifications may indeed prove possible, they will often come at a price that is higher than the costs of modifying our environment, while being no more autonomy respecting than the proposals I advance.

A central reason why environmental engineering will often be preferable to modification of our onboard capacities is that the biases and volitional pathologies we aim to correct are often generated by modules or processes that also produce significant benefits. Indeed, the benefits of at least some of the relevant adaptations are so significant that some cognitive scientists believe that on balance they continue to be beneficial in the current environment (Gigerenzer et al. 1999). Hedonic adaptation, for instance, may make us bad affective forecasters, but also enables us to better cope with misfortune—surely an important function, given that we all face disease and death. The benefits of our pervasive overconfidence have already been mentioned: it helps to motivate us to perform at our peak capacity. In general, fast and frugal processing of the kind that underlies many of our characteristic cognitive weaknesses is adaptive because it allows us to automate our responses, thereby preserving cognitive resources for novel and difficult tasks. Indeed, given that our current environments place cognitive demands on us that are novel, reliance on such frugal processing mechanisms may today be even more necessary than it was in the environment of evolutionary adaptiveness in which these mechanisms evolved. Today more than ever, we rely on fast and frugal processes to reduce our decision space to tractably many options. Modifying ourselves so that we no longer had the disposition to engage such processes seems likely to impose very serious costs on ourselves, making decision making unacceptably slow and demanding without a significant compensatory gain in accuracy.

Might it be possible to modify ourselves so that we retain the benefits of heuristics and biases without the costs? While this may sometimes be possible, in many cases it will prove very difficult for reasons to do both with the function of the relevant mechanisms and with their implementation. Functionally, these mechanisms cannot discriminate between circumstances that call for fast and frugal processing and those that call for slower and more effortful processing: making such discriminations typically *requires* engaging in effortful processing. Engage in such processing and the benefits of fast and frugal mechanisms are *already* lost. Further, the relevant mechanisms may often be causally linked to an enormous range of functions. Similarly, the neurotransmitters that might be pharmaceutically altered are likely to be involved in a broad range of functions. Serotonin, for instance, augmentation of which increases social affiliative behavior (Tse and Bond 2002, 2003), is also involved in cardiovascular regulation, respiration, sleep–wake cycles and also appetite, pain sensitivity, and reward learning (Churchland 2011: 98). Even within the domain of (say) morality, functions seem to dissociate in various ways, such that enhancing one leads to decrements in others. For example, enhancements of elements of moral cognition using selective serotonin reuptake inhibitors come at the cost of an increased willingness to allow cheaters to go unpunished (Crockett et al. 2010).

Beyond the domain of morality, enhancements of function also seem to regularly cause decrements in other functions. For instance, Wei et al. (2001) enhanced aspects of memory in mice, but the genetic modification also resulted in much higher sensitivity to pain. Moreover, it has long been known that human beings with prodigious memories often have trouble with abstraction, presumably because recall of detail interferes with focus on the most significant aspects of a situation (Liao and Sandberg 2008). Even when a function is subserved by a module—a dissociable, functionally discrete, system dedicated to a single domain—there are good reasons to think that modifications aimed at altering the module will have effects on other cognitive processes. Modules may frequently share components with other modules, in order to minimize the energetic costs of resource-hungry cognitive processes (Carruthers 2006).

It may nevertheless sometimes be possible to modify our onboard capacities in ways that reduce their costs without causing further problems. For instance, we might have dispositions—our liking for high-calorie, high-fat foods springs to mind—that were once adaptive but which now bring with them no significant benefit; these dispositions may be apt for modification (see Powell and Buchanan 2011 for discussion). Even in many of these cases, however, it may prove much cheaper to alter the environment to avoid triggering the disposition than to engage in high-tech manipulations. An environmental manipulation—altering the opening hours of bottle shops, say—may be effective for many thousands of individuals at once, whereas modifications of onboard capacities may have to be on an individual by individual basis. It may sometimes prove possible to modify onboard capacities cheaply (supplementation with iodine is a familiar example; moreover, one that plausibly counts as an enhancement; see Levy 2011b), but large-scale use of these kinds of modifications will often be prohibitively expensive.

## Conclusion: Democratic Social Engineering

These methods of circumventing problems with affective forecasting and hyperbolic discounting either restrict the options of agents tout court or make some options more costly, thereby effectively restricting them. Prima facie, then, these are paternalistic measures that infringe liberal notions of autonomy. However, there are several reasons to doubt that they are genuinely paternalistic; at very least, if paternalism is understood as infringing autonomy for the agent’s own goods, these interventions are not paternalistic.

Importantly, if we are less influenced by our affective forecasts or less subject to mental contamination, we will be better decision makers, by *our own* lights. Affective forecasting leads to ill-informed decisions: decisions that are less likely to lead to ends we value. If the principle justification of liberal societies is that they are the form of political organization that allows each of us to pursue our own conception of the good without undue interference, then measures that make us better at achieving our own ends are not unacceptably paternalistic. Importantly, they are not measures justified by a welfare criterion, like Loewenstein and Haisley’s (2008) “light paternalism.” Rather, they are justified by the extent to which they enable agents effectively to pursue goals they antecedently endorse.

There are, however, objections to these kinds of measures independent of worries about whether they infringe autonomy. At least two remaining objections date back to the Enlightenment itself. In addition to its optimism about the power of human reason, Enlightenment opposition to paternalism received support from a belief in the intrinsic value of the exercise of autonomy, and from a worry that those in positions in power cannot be trusted to identify and act in the interests of others, rather than themselves. Both these objections have their proponents today. For instance, some contemporary thinkers worry that reducing the scope for agents to make mistakes is itself objectionable, inasmuch as full human development requires making mistakes and learning from them (e.g., Klick and Metchell 2006). In response, it is worth making two points. First, no proposal that could conceivably be justified as public policy will significantly reduce the sheer number of opportunities individuals have for making mistakes and—hopefully—learning from them. Public policy measures are aimed at widely shared problems; each individual, as he or she pursues his or her own life and goals, will confront innumerable problems unique to their situation, and will therefore have plenty of scope for making mistakes. Second, the kinds of problems that these proposals address are often problems to which many, perhaps most, individuals seem incapable of learning responses that do not involve self-binding in the kinds of ways proposed. This is the case for several reasons, two prominent among them: because the resources we rely upon to control our behavior in the face of temptation are depletable (Levy 2011a) and because the problems of abundance are evolutionarily novel, and therefore we have no evolved onboard capacities for solving them.

The second worry about paternalism or managerialism is rooted in a distrust of authorities, whether kings or technocrats, to act in the interests of those they ostensibly serve, rather than in their own interests. This is a sensible worry. In response, however, it is worth making three points. First, worries like this arise acutely when there is a conflict between the interests of the governed and the governing. This need not be the case with regard to the proposals suggested here. Governments may have an interest in encouraging consumption of the kinds of goods that agents find difficult to resist, like alcohol and tobacco, because these goods are traditional sources of tax revenue (perhaps because indulgence is seen as a vice and therefore as appropriately taxed). But governments also have an interest in reducing consumption of these same goods since both directly (through burdens on the healthcare system) and indirectly (through a reduction in economic productivity) these behaviors hit tax revenues. It is generally held by economists that the costs of consumption to governments are substantially higher than the revenue recouped (Ahmad and Franz 2008). There is therefore no conflict between the interests of the governed and the governing here. Similar remarks apply to proposals aimed at boosting savings for retirement. Worries like this one, which turns on a conflict between the interests of the governed and the governing, are more pressing for proposals that countenance measures to change the values and beliefs of people: proposals like those advanced here, which utilize these values and the deeply held beliefs of people as a test for the permissibility of interventions, raise these worries far less. That fact brings us immediately to the second point, which is that these measures are and ought to be open to democratic review. These measures can be endorsed by us, in a cool hour, when we see that they allow us to make choices that are in line with our own values. These are measures of a kind that agents can reasonably choose to impose on ourselves (for instance, by putting time locks on drinks cabinets); prima facie they are also measures we can impose on ourselves as a society. Measures like these can be implemented democratically: they can be openly debated and imposed by accountable governments.

The third response to distrust of elected officials consists in pointing out that we do not face a choice over *whether* our knowledge of our psychological dispositions will be put to work in affecting our behavior; the choice concerns *who* will utilize this knowledge. To refuse to allow governments to utilize the knowledge is to turn the field over to private industry, and we can be more confident of conflicts of interest between industry and consumers than between democratically elected governments and electorate. Of course, we may legitimately ask governments to regulate the behavior of third parties without asking it to regulate our own behavior. However, that is and ought to be a matter for democratic decision.

There are therefore no good objections from autonomy to ecological engineering of the kind envisaged here. Rather than infringing on our autonomy, the measures envisaged may rather be seen as enhancing it. There are strong grounds for employing a range of techniques to burden unwise choices and to encourage wise choices, when we can do so without actually blocking any choices, should anyone be genuinely committed to making them. Human beings are inveterate ecological engineers, reshaping the natural and social worlds to enhance our well-being. We already apply our scientific knowledge to allow us to achieve goals that are valuable for us, that promote our flourishing. We should apply our social scientific knowledge in similar ways. We need to develop a technology of social shaping, creating our social niches to better allow us to achieve eudemonia.^4^

## Footnotes

1 As a reviewer for this journal points out, Enlightenment opposition to paternalism had additional sources as well as faith in reason. Some currents in Enlightenment thought also emphasized the value of autonomous choice, and some also expressed distrust of government to make choices that were genuinely in the interests of the governed. These are important objections to paternalistic interventions, as well as to the interventions I shall sketch. I shall briefly address these concerns in the concluding section of this paper.

2 Popper (1961) famously distinguished between piecemeal and utopian social engineering: whereas the former seeks to better design institutions to allow us to achieve our goals, the latter seeks to remake society from the ground up. Popper predicted that utopian social engineering would lead to despotism and the widespread violation of fundamental human rights. Any proposal that takes people as they are, with their goals and beliefs, and tests the permissibility of social engineering against these attitudes, will likely be piecemeal and not utopian, and the proposals I will advance certainly fit the bill.

3 The assumption that people aim at happiness is characteristic of modernity; see McMahon (2010) for discussion. Subjects in experiments readily report strategies they utilize to increase their happiness, suggesting that they take themselves to pursue it.

4 I would like to thank Russell Powell and two anonymous reviewers for *Philosophy and Technology* for comments that have improved almost every aspect of this paper. I gratefully acknowledge the support of the Australian Research Council.
